# Liver saturated fat content associates with hepatic DNA methylation in obese individuals

**DOI:** 10.1186/s13148-023-01431-x

**Published:** 2023-02-11

**Authors:** Ratika Sehgal, Alexander Perfilyev, Ville Männistö, Jyrki Ågren, Emma Nilsson, Pirjo Käkelä, Charlotte Ling, Vanessa D. de Mello, Jussi Pihlajamäki

**Affiliations:** 1 Department of Clinical Nutrition, Institute of Public Health and Clinical Nutrition, University of Eastern Finland, Kuopio, Finland; 2 Epigenetics and Diabetes Unit, Department of Clinical Sciences, Lund University Diabetes Centre, Scania University Hospital, Malmö, Sweden; 3 Department of Medicine, University of Eastern Finland and Kuopio University Hospital, Kuopio, Finland; 4 Institute of Biomedicine, School of Medicine, University of Eastern Finland, Kuopio, Finland; 5 Department of Surgery, University of Eastern Finland and Kuopio University Hospital, Kuopio, Finland; 6 Department of Medicine, Endocrinology and Clinical Nutrition, Kuopio University Hospital, Kuopio, Finland

**Keywords:** Saturated fatty acids, NAFLD, DNA methylation, Obesity, Fasting plasma glucose, Epigenetics, Lipids

## Abstract

**Background:**

Accumulation of saturated fatty acids (SFAs) in the liver is known to induce hepatic steatosis and inflammation causing non-alcoholic fatty liver disease (NAFLD) and non-alcoholic steatohepatitis (NASH). Although SFAs have been shown to affect the epigenome in whole blood, pancreatic islets, and adipose tissue in humans, and genome-wide DNA methylation studies have linked epigenetic changes to NAFLD and NASH, studies focusing on the association of SFAs and DNA methylation in human liver are missing. We, therefore, investigated whether human liver SFA content associates with DNA methylation and tested if SFA-linked alterations in DNA methylation associate with NAFLD-related clinical phenotypes in obese individuals.

**Results:**

We identified DNA methylation (Infinium HumanMethylation450 BeadChip) of 3169 CpGs to be associated with liver total SFA content (*q*-value < 0.05) measured using proton NMR spectroscopy in participants of the Kuopio Obesity Surgery Study (*n* = 51; mean ± SD:49.3 ± 8.5 years old; BMI:43.7 ± 6.2 kg/m^2^). Of these 3169 sites, 797 overlapped with previously published NASH-associated CpGs (NASH-SFA), while 2372 CpGs were exclusively associated with SFA (Only-SFA). The corresponding annotated genes of these only-SFA CpGs were found to be enriched in pathways linked to satiety and hunger. Among the 54 genes mapping to these enriched pathways, DNA methylation of CpGs mapping to *PRKCA* and *TSPO* correlated with their own mRNA expression (HumanHT-12 Expression BeadChip). In addition, DNA methylation of another ten of these CpGs correlated with the mRNA expression of their neighboring genes (*p* value < 0.05). The proportion of CpGs demonstrating a correlation of DNA methylation with plasma glucose was higher in NASH-SFA and only-SFA groups, while the proportion of significant correlations with plasma insulin was higher in only-NASH and NASH-SFA groups as compared to all CpGs on the Illumina 450 K array (Illumina, San Diego, CA, USA).

**Conclusions:**

Our results suggest that one of the mechanisms how SFA could contribute to metabolic dysregulation in NAFLD is at the level of DNA methylation. We further propose that liver SFA-related DNA methylation profile may contribute more to hyperglycemia, while insulin-related methylation profile is more linked to NAFLD or NASH. Further research is needed to elucidate the molecular mechanisms behind these observations.

**Supplementary Information:**

The online version contains supplementary material available at 10.1186/s13148-023-01431-x.

## Background

Non-alcoholic fatty liver disease (NAFLD) is the most common chronic liver disease that spans in severity from simple steatosis to non-alcoholic steatohepatitis (NASH) and, ultimately, to hepatocellular carcinoma [1]. Excessive intake of dietary saturated fatty acids (SFAs), increased free FAs (FFAs) released into the circulation from adipose tissue, upregulated de novo lipogenesis in the liver, and production of new hepatic fatty acids from carbohydrates or proteins are all known to induce hepatic steatosis and inflammation, causing NAFLD and NASH [2–5].

The lipotoxic effects of FFAs such as stress, autophagy, lipoapoptosis, and inflammation resulting from an imbalance in the hepatic FFAs availability and disposal are aggravated by the SFAs [6–8]. The largest contributor to the accumulation of hepatic triglycerides (TG) is saturated fats [9]. An excessive accumulation of SFAs in liver TG in individuals with non-alcoholic fatty liver or NASH compared to the normal liver has been recently reported [10]. Moreover, evidence suggests that the increased hepatic SFAs levels are linked to the cellular, oxidative, endoplasmic reticulum, and mitochondrial stress in NAFLD [11–14]. In fact, based on in vitro and in vivo studies, overexposure of SFAs in the cell culture medium or in the diet leads to inflammation, altered insulin signaling, and apoptosis in liver cells [15–17].

The pathogenesis of NAFLD is known to be modifiable by lifestyle factors and genetic variations [18], both of which may contribute to epigenetic dysregulation in NAFLD [19–21]. At present, the most widely and extensively investigated epigenetic modification, specifically in metabolic diseases, is DNA methylation [22]. It is a reversible change playing a critical role in regulation of transcription, embryonic development, genomic imprinting, and chromatin structure [23]. These DNA methylome changes are often tissue specific and play a crucial role in reprogramming the cellular machinery as an adaptive response to calorie-excess environments, as in the case of NAFLD [22, 24].

Many lipids, including FFAs and SFAs, have been proposed to cause NAFLD-linked epigenetic changes [25]. More specifically, SFA-induced changes in DNA methylation have been proposed in cultured human pancreatic islets and human adipose tissue. Furthermore, whole blood DNA methylation has been associated with dietary intake of fat in cross-sectional epidemiological studies carried out in normal-weight and obese children [26–29]. However, whether liver SFAs could influence the whole liver methylome has never been explored. Thus, the aim of the present study was to identify liver DNA methylation patterns that associate with hepatic SFAs content in obese individuals. In addition, we sought to explore whether these alterations in SFA-related DNA methylation associate with NAFLD-related clinical phenotypes.

## Results

### Liver total saturated fat content is elevated in NAFLD

The clinical characteristics of the Kuopio Obesity Surgery (KOBS) study participants categorized based on their liver histology are shown in Table 1. Fasting serum triglycerides (TG) and insulin levels were significantly different across the three groups: normal liver, simple steatosis (SS), and non-alcoholic steatohepatitis (NASH) (*p* value < 0.05). The total liver saturated fat content (SFA, See Table 1) was significantly elevated in those with SS and NASH as compared to those with normal liver (*p* value < 0.05). However, the saturated fat content was not different between the SS and NASH groups.

**Table 1:** Clinical characteristics and liver histology of study participants according to histological liver phenotype

	Normal liver	SS	NASH	*p* value^a^
Total, *n*	24	12	15	–
Men/Women, *n*	8/16	5/7	6/9	n.s.
Age (years)	49.6 ± 8.3	47.3 ± 8.2	50.3 ± 9.2	n.s.
BMI (Kg/m^2^)	43.0 ± 6.3	44.4 ± 5.5	44.1 ± 6.6	n.s.
fS-Total cholesterol (mmol/L)	4.1 ± 0.7	4.0 ± 0.9	4.1 ± 0.9	n.s.
fS-LDL-c (mmol/L)	2.4 ± 0.7	2.1 ± 0.7	2.4 ± 0.7	n.s.
fS-HDL-c (mmol/L)	1.0 ± 0.2	0.9 ± 0.1	1.0 ± 0.2	n.s.
fS-TG (mmol/L)	1.2 (1.0–1.6)	1.8 (1.3–2.3)*	1.3 (1.0–1.9)	0.027
fP-Glucose (mmol/L)	5.8 ± 0.6	6.0 ± 1.2	6.7 ± 1.6	n.s.
fS-Insulin (mU/L)	12.9 (7.6–19.2)	14.9 (10.0–21.8)	15.8 (12.6–28)*	0.041
Type 2 diabetes (N/Y), n	18/6	6/6	6/9	n.s.
Total liver SFA content	61.7 ± 19.7	101.0 ± 34.2*	123.9 ± 65.5*	0.00002

Data are shown as mean ± SD or median (IQR). fS—Fasting serum; fP—fasting plasma; LDL-c—low-density lipoprotein cholesterol; HDL-c—high-density lipoprotein cholesterol; TG—triglycerides; N/Y—no/yes; n—number of individuals; and SFA—saturated fatty acids. ^a^One-way ANOVA test (continuous variable) or *χ*^2^ test (categorical variable) over the three study groups; post hoc Bonferroni correction was used for multiple testing, *p value < 0.05 versus normal liver

### Identification of SFA-related DNA methylation in human liver

**Fig. 1:**
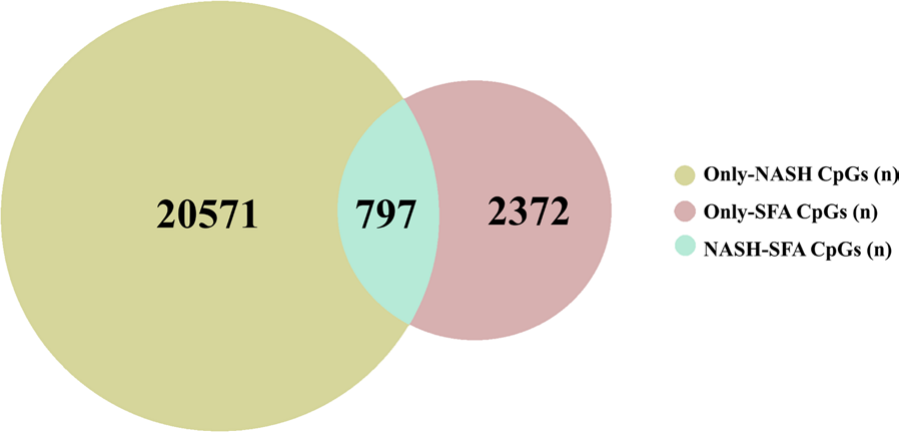
Overlap between the NASH-related and SFA-related CpGs in human liver. The Venn diagram represents the number of CpGs belonging to specific only-NASH and only-SFA groups and to the ones overlapping NASH-SFA group. The NASH-related CpG sites were defined based on our previous publication [30], and the SFA-related CpG sites were identified as described in the results

We found that DNA methylation of 3169 CpG sites (CpGs), representing 1881 unique genes, was significantly associated with liver SFA at a false discovery rate (FDR) below 5% (*q*-value < 0.05, adjusted for gender, body mass index (BMI), and age; Additional file 1: Table S1). Methylation on five of these 3169 sites was significantly associated with liver SFA after Bonferroni correction (Additional file 1: Table S1). Data from all the 51 individuals (Normal liver + SS + NASH) were combined for this analysis. We have previously shown that DNA methylation of 21,368 CpGs, representing 7788 unique genes, was associated with NASH [30]. In order to find the DNA methylation changes exclusively associated to SFA, we identified the CpGs specifically associated to SFA and not to NASH. In Fig. 1, we describe that among the 3169 CpGs associated with liver SFA, 797 overlapped with those 21,368 that were previously observed to be NASH-related (termed NASH-SFA). Consequently, the remaining 2372 SFA-related CpGs, representing 1424 unique genes, were exclusively related to liver SFA (Only-SFA). Out of the 21,368 NASH-related CpGs [30], 20,571 were exclusively related to NASH (Only-NASH). From now on, we will address these as three groups named as follows, only-NASH, NASH-SFA, and only-SFA (Fig. 1).

### Genomic location enrichment for the identified CpGs

**Fig. 2:**
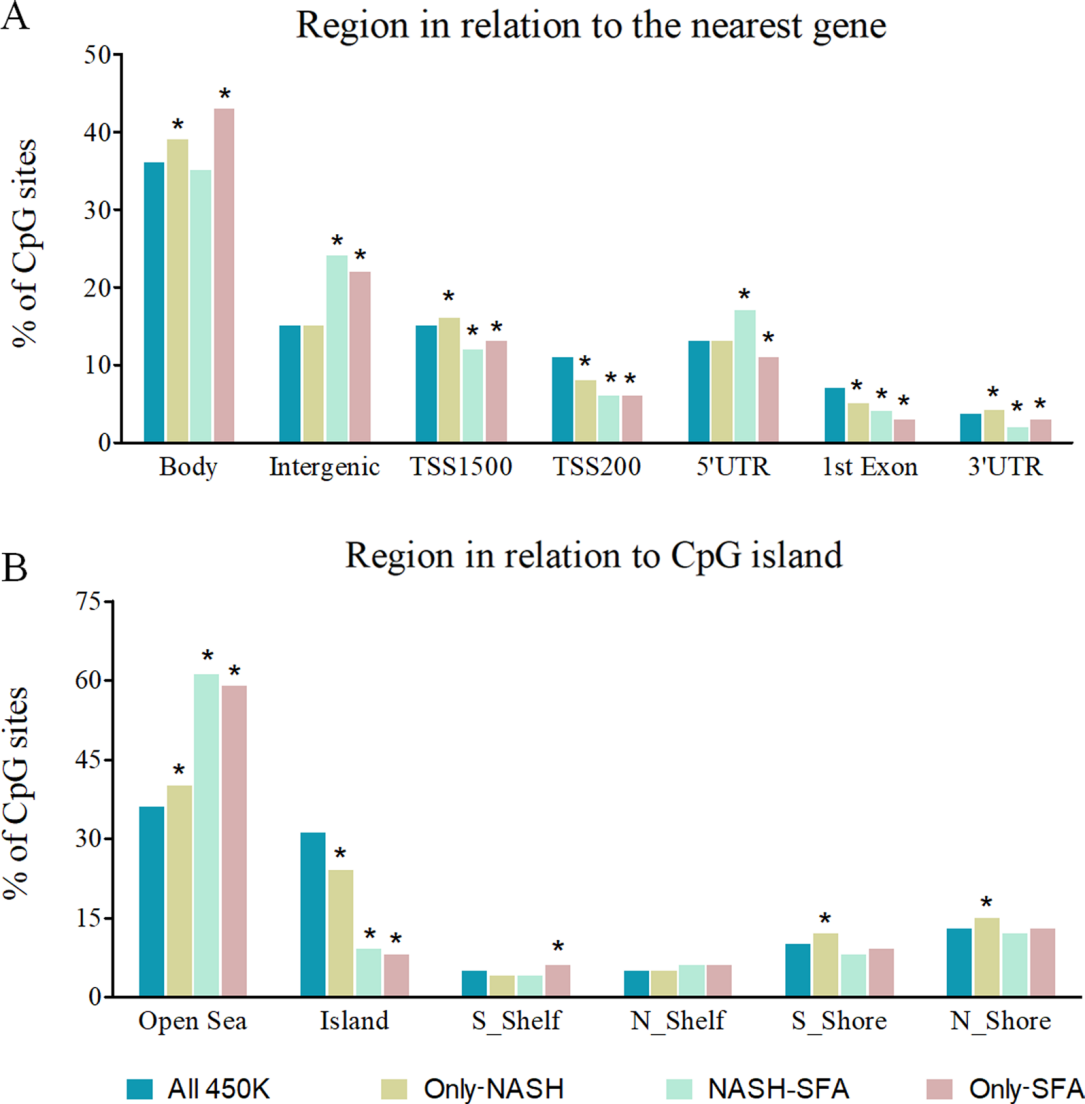
Genomic location enrichment for CpGs associated with SFA and/or NASH in human liver. Percentage of all CpGs located in relation to (**A**) nearest gene regions and (**B**) CpG island having important implications for the regulation of gene expression for only-NASH, NASH-SFA, and only-SFA groups, with percentage of all 450 K CpGs as control. CpG islands were defined as DNA sequences (as 500 base windows; excluding most repetitive Alu-elements) with a GC base composition greater than 50% and a CpG observed ratio [31] of more than 0.6. TSS1500-region within 1500 base pair upstream of a transcription start site; TSS200-region within 200 base pair upstream of a TSS; UTR-Untranslated region; N_Shore-2 kb regions upstream of CpG island; S_Shore-2 kb regions downstream of CpG island; N_Shelf-2 kb regions upstream of CpG island shore; S_Shelf-2 kb regions downstream of CpG island shore; and Open Sea-regions > 4 kb from CpG islands. *Indicates that the proportion of CpGs in only-NASH (*n* = 20,571), NASH-SFA (*n* = 797), and only-SFA (*n* = 2372) was significantly (*p* value < 0.01) different compared to all sites covered on the 450 K BeadChip (All 450 K), (*χ*^2^ test of independence with 1° of freedom). Each bar represents % of sites

The genomic location enrichment for all the significant CpGs in each group (Only-NASH, NASH-SFA, and only-SFA) based on gene region showed significant differences between groups (Fig. 2A). For all the three groups of CpGs, the largest proportion of CpGs was localized in the gene bodies and the intergenic regions. Further upstream (TSS1500), NASH-SFA and only-SFA groups were found to have significantly reduced proportions, while for only-NASH the proportions were higher compared to all 450 K array. For all the near gene transcription starting points (TSS200, 5′UTR, and 1st Exon), among the three groups, the proportions of only-SFA associated CpGs were lower compared to CpG on the whole 450 K array. In fact, the proportions were also found to be reduced for only-NASH and NASH-SFA for the TSS200 and 1st Exon regions, when compared to all the whole 450 K array. However, for 5′UTR region, proportions of CpGs were found to be significantly increased in NASH-SFA group. At the end region of the genes (3′UTR), all the three groups followed a similar trend as in the upstream region: TSS1500 (Fig. 2A). The percentage of CpGs based on island functional categories shows significant overrepresentation of CpGs for all the three groups in the open sea and a significant underrepresentation in the CpG islands (Fig. 2B). Also, only-SFA associated CpGs were significantly more localized in the southern shelf region (2-4 kb from CpG islands).

### Pathway analyses for the genes annotated to CpG sites

To better elucidate the biological mechanisms of the significantly associated CpGs with SFA, we applied pathway analyses to the annotated genes that were associated with only-SFA and NASH-SFA. As illustrated in Table 2, annotated genes to only-SFA were enriched for pathways related to morphine addiction, neuroactive ligand–receptor interaction, and retrograde endocannabinoid signaling. In contrast, NASH-SFA genes were significantly enriched for pathways related to cancer, inflammatory response, and insulin signaling.

**Table 2:** Pathway enrichment for the NASH-SFA- and only-SFA-associated genes

	Pathways	Gene overlap	*q*-value
Only-SFA	Morphine addiction	18/91	0.012
	Retrograde endocannabinoid signaling	19/101	0.012
	Neuroactive ligand–receptor interaction	36/278	0.034
NASH-SFA	Pathways in cancer	25/397	0.0031
	EGFR tyrosine kinase inhibitor resistance	9/81	0.022
	Melanoma	8/71	0.031
	Endocytosis	16/260	0.032
	PI3K-Akt signaling pathway	19/341	0.032
	Rap1 signaling pathway	14/212	0.032

The pathways shown above are Kyoto Encyclopedia of Genes and Genomes (KEGG) pathways using the WebGestalt tool. The gene overlap means the number of genes identified in our dataset/total number of genes known to be involved in a particular pathway. *q*-value (FDR) for each pathway is shown in the last column. EGFR—Epidermal growth factor receptor; PI3K-Akt—phosphatidylinositol 3‑kinase/protein kinase B; and Rap1-Ras-proximate-1.

### Interaction mapping for only-SFA annotated genes

**Fig. 3:**
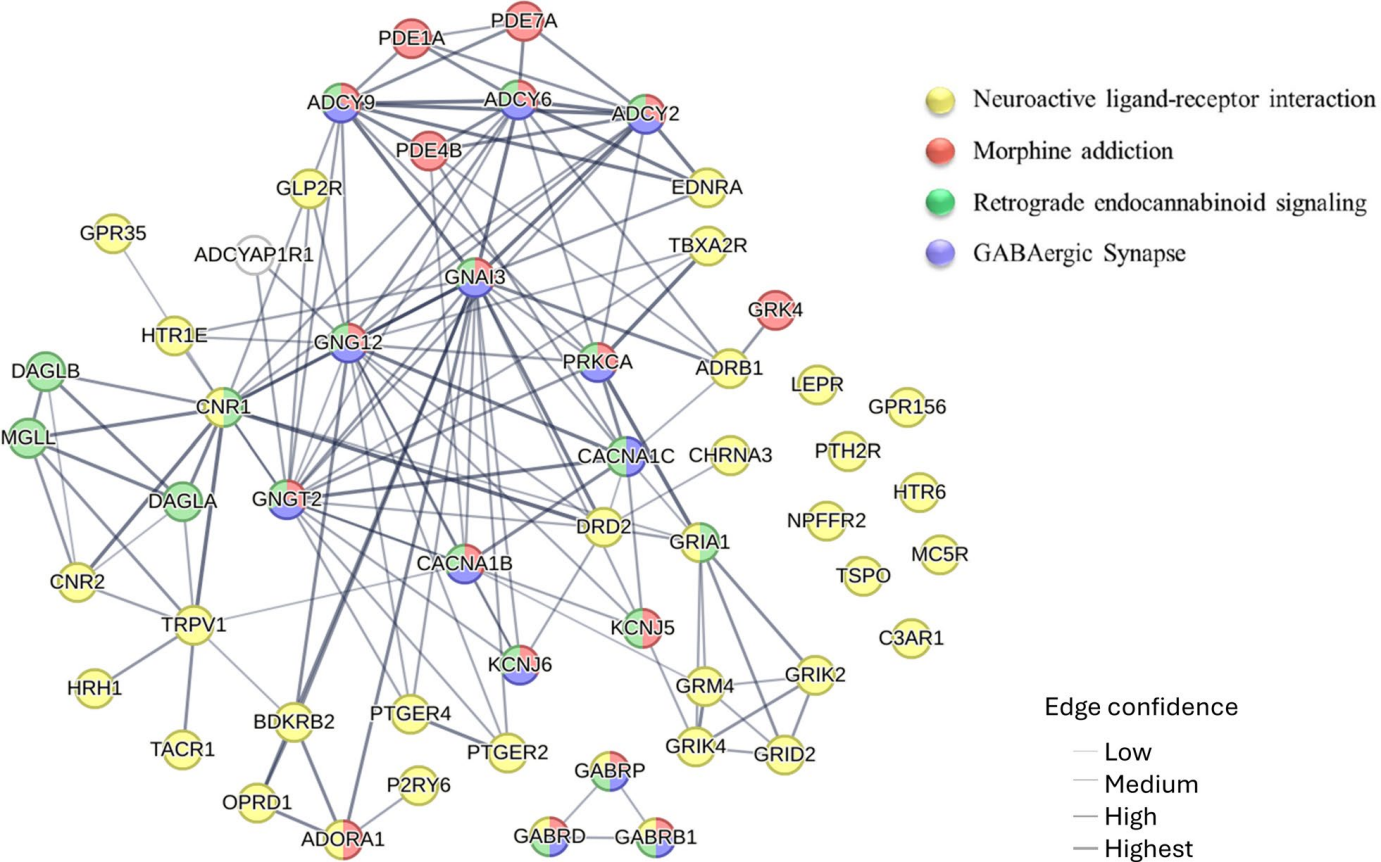
Interaction map of 54 genes corresponding to the enriched KEGG pathways for only-SFA. The network nodes represent proteins and edges represent protein–protein associations. The genes with maximum interactions or involved in all four pathways are marked with a black star. The line thickness is the edge confidence that indicates the strength of data support (based on homology, co-expression, experimentally determined interactions, database annotations, and automated text mining). The edge confidence represented by the thickness of the connecting lines is categorized into four levels, low, medium, high, and highest

A total of 54 genes corresponding to the only-SFA enriched pathways (Table 2) were used as an input to build an interaction map using the StringDB online tool [32]. Based on the interaction confidence level (edge confidence), the topmost interactive genes were: *CACNA1B* (calcium voltage-gated channel subunit alpha1 B), *CNR1* (cannabinoid receptor 1), *GNAI3* (guanine nucleotide-binding protein [G Protein], alpha-inhibiting activity polypeptide 3), *PRKCA* (protein kinase C alpha), *GNGT2* (guanine nucleotide-binding protein G(I)/G(S)/G(O) subunit gamma-T2), *GNG12* (guanine nucleotide-binding protein G(I)/G(S)/G(O) subunit gamma-12), *ADCY6* (adenylate cyclase type 6), and *DRD2* (dopamine receptor D2). From these genes, those that were also found to be enriched in more than one pathway are marked with a star in Fig. 3. In addition to the KEGG pathways analysis, we found three *GABA*-related genes to be involved in all the four enriched pathways, *GABRB1* (gamma-aminobutyric acid type A receptor subunit beta1), *GABRD* (gamma-aminobutyric acid type A receptor subunit delta), and *GABRP* (gamma-aminobutyric acid type A receptor subunit Pi).

### SFA-related DNA methylation alterations correlate with gene expression

The same set of CpGs related to only-SFA (Table 2 and Fig. 3) were also tested for their correlation of DNA methylation with the mRNA expression. Out of 64 CpGs (54 annotated genes), gene expression data were available for 14 genes on the Illumina expression array. We found the gene expression of *PRKCA* and *TSPO* (Translocator Protein) to be associated with the methylation of the corresponding CpG site with a *p* value < 0.05 (*PRKCA*-cg14648237 and *TSPO*-cg13160331, Table 3). Alternatively, we correlated DNA methylation of all the 64 CpGs with the expression for transcripts located in the genomic region around these CpGs (within the *cis* distance 500 kb upstream and 100 kb downstream of the gene; Additional file 5: Table S2) and identified ten significant correlations between the DNA methylation of the CpGs and mRNA expression (*p* value < 0.05, Table 3). Among these, the strongest correlation was the one between methylation of cg11821200 and mRNA expression of *PRKAA1* (protein kinase AMP-activated catalytic subunit alpha 1). We also found correlations between DNA methylation of cg07011711 and cg00437258 with the mRNA expression of *SDHAF2* (succinate dehydrogenase complex assembly factor 2) and *RAC1* (Rac family small GTPase 1), respectively (Table 3).

**Table 3:** Pearson correlations (*r*) between DNA methylation and mRNA expression at *p* value < 0.05

	CpG ID^1^	Transcript ID^2^	*r*	*p* value
Gene expression (self)	cg14648237	*PRKCA*	0.585	0.0066*
	cg13160331	*TSPO*	− 0.537	0.0145
Gene expression (neighboring)	cg11821200	*PRKAA1*	0.274	0.0005*
	cg00437258	*RAC1*	− 0.565	0.0093
	cg07011711	*SDHAF2*	− 0.547	0.0124
	cg15892963	*C14orf132*	0.527	0.0168
	cg24191821	*FCHSD2*	0.504	0.0232
	cg19678564	*KDM5B*	0.468	0.0401
	cg26151675	*GALE*	0.461	0.0407
	cg14109579	*DUSP28*	− 0.459	0.0416
	cg00437258	*ZDHHC4*	0.449	0.0465
	cg18274619	*ABTB1*	-0.445	0.0490

^1^Infinium HumanMethylation450 BeadChip-based DNA methylation and ^2^HumanHT-12 Expression BeadChip-based mRNA expression. *PRKCA*—Protein kinase C alpha; *PRKAA1*—protein kinase AMP-activated catalytic subunit alpha 1; *TSPO*—translocator protein; *SDHAF2*—succinate dehydrogenase complex assembly factor 2; *RAC1*—Rac family small GTPase 1; *DUSP28*—dual specificity phosphatase 28; *ABTB1*—ankyrin repeat and BTB domain-containing 1; *C14orf132*—chromosome 14 open reading frame 132; *FCHSD2*—FCH and double SH3 domains 2; *GALE*—UDP-galactose-4-epimerase; *ZDHHC4*—zinc finger DHHC-type containing 4; and *KDM5B*—lysine demethylase 5B. The Pearson correlation test was used for the analysis, and *p* value < 0.05 was considered statistically significant. *p value < 0.1 (adjusted for multiple testing)

### Correlation of SFA-related DNA methylation of CpGs with clinical variables

**Fig. 4:**
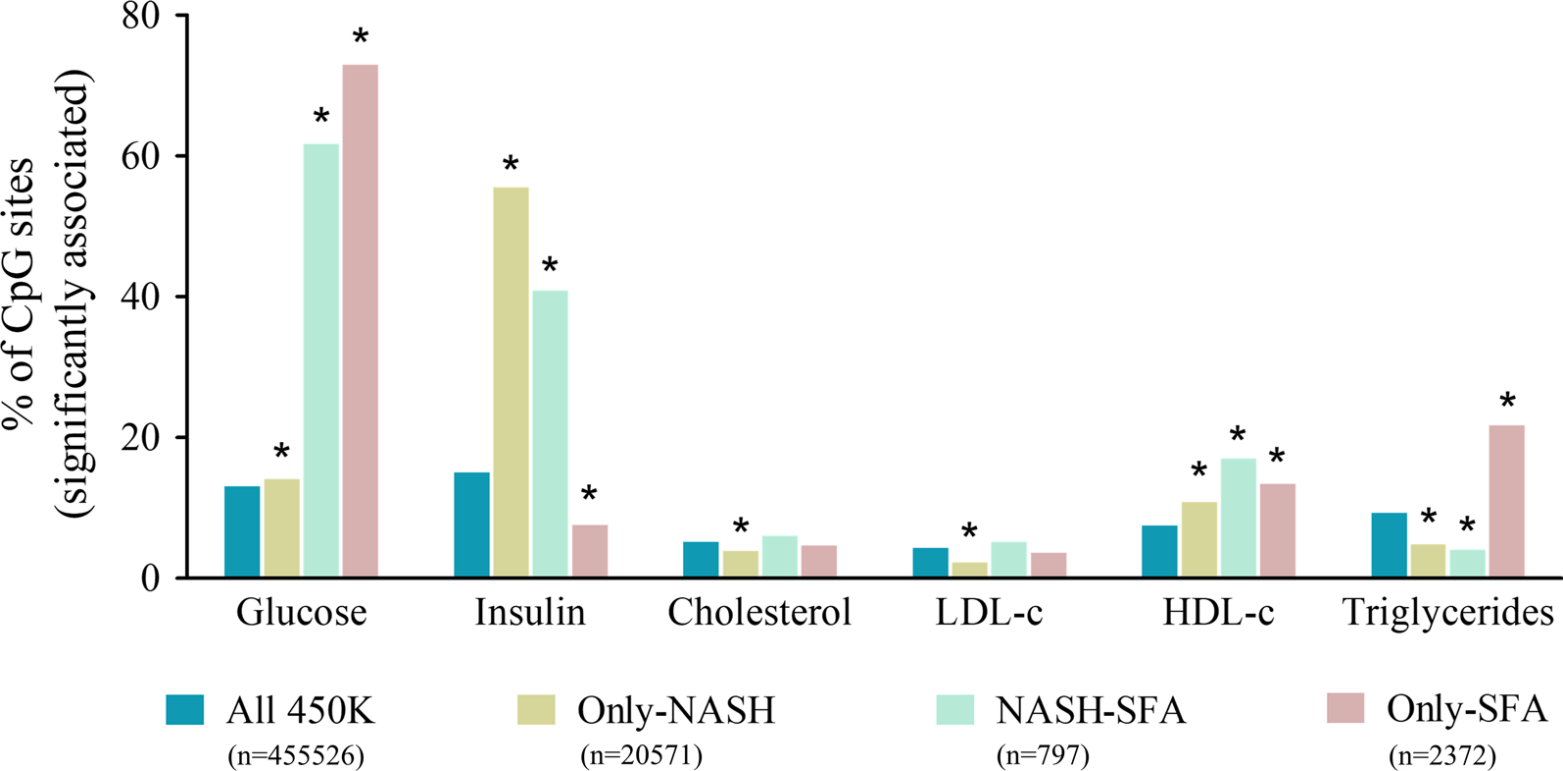
Bar plot representing the percentage of CpG sites associated significantly with each of the clinical variables. For each of the clinical variables (glucose, insulin, total cholesterol, LDL-c, HDL-c, and TG), the proportions of the significantly (*p* value < 0.05) correlated CpGs for four groups defined in the key were analyzed. *Indicates the proportion of sites significantly (*p* value < 0.05) different compared to the proportions for the 450 K BeadChip (All 450 K) for each clinical variable, tested using the *χ*^2^ test of independence with 1° of freedom

In an effort to decipher the clinical significance, we tested the correlations of DNA methylation of the three groups of CpGs (Only-NASH, NASH-SFA, and only-SFA) with the laboratory measurements reflecting lipid and glucose metabolism: serum lipids, serum insulin, and plasma glucose levels (represented as a heatmap in Additional file 2: Fig. S1; adjusted *p* value < 0.1 tabulated in Additional file 6: Table S3). Using these data, we calculated the proportions of significantly correlated CpGs in each of these groups compared to the proportion of significantly correlated CpGs in the whole 450 K BeadChip array. The proportions of significantly correlated CpGs were found to be statistically significantly higher for fasting plasma glucose, fasting serum insulin, and fasting serum TG in only-SFA group when compared to the proportions in the whole 450 K array (*p* value < 0.05, Fig. 4). For plasma glucose levels, the proportions of significantly correlated CpGs were found to be increased for all three groups, when compared to all 450 K. Interestingly, for serum insulin levels the proportions of correlated CpGs for only-SFA were found to be significantly lower as compared to only-NASH and NASH-SFA groups, where the proportions were found to be higher compared to the whole array. An opposite trend was observed for the TG levels, with significantly higher proportions of correlated CpGs for only-SFA and lower proportions for only-NASH and NASH-SFA, compared to the whole 450 K. All these comparisons were also significant (*p* value < 0.05) when adjusted *p* value was used for the correlations.

### Correlation of individual SFAs with the SFA-related DNA methylation

Next, we checked if individual SFAs (14:00-myristic acid, 15:00-pentadecanoic acid, 16:00-palmitic acid, 17:00-heptadecanoic acid, and 18:00-stearic acid) correlate distinctly with SFA-related DNA methylation. The data demonstrating correlations of these individual SFAs proportions (mol%) with the DNA methylation of the CpGs within each of the groups (only-NASH, NASH-SFA, and only-SFA) are shown in Additional file 3: Fig. S2. The correlation analysis was also adjusted for multiple testing (*p* value < 0.1), and the proportions were calculated across the groups (Additional file 7: Table S4). We found that palmitic and stearic acid correlated more often with DNA methylation of the CpGs linked with SFA as compared to other SFAs in NASH-SFA and only-SFA groups.

## Discussion

In the present study, we observed that liver content of total saturated fat (SFA) associates with the DNA methylation profile in the liver of obese individuals, suggesting that SFA may regulate hepatic DNA methylation. When excluding those CpGs that we previously identified to be NASH-related [30], we found that the CpGs solely methylated in relation to liver SFA were enriched for pathways modulating satiety and food intake. These results were supported by the findings that methylation in some of these CpGs correlated with the expression of genes annotated to these CpGs, or with the expression of neighboring genes. The observed differences in correlations of the DNA methylation of these with SFA- and NASH-related CpGs with fasting plasma glucose and serum insulin levels may reveal specific mechanisms related to NASH-linked clinical phenotypes.

To our knowledge, this is the first study that attempts to identify SFA-related DNA methylation changes in the human liver. We identified total liver saturated fat content measured using proton nuclear magnetic resonance (NMR), to be associated with DNA methylation of 3169 CpGs in the current study (Fig. 1). It is well known that epigenetics plays an important part in the development of NAFLD and is often considered a modifiable contributing factor [19, 21, 33]. Earlier studies carried out in humans have reported SFA-related DNA methylation changes in whole peripheral blood, adipose tissue, and cultured pancreatic islets [27–29]. Many of the identified CpG methylation sites in these studies were found to be linked with glucose and lipid metabolism, in line with the findings in this study. Furthermore, in vitro and in vivo studies have highlighted the detrimental effects of SFAs on mechanisms linked with inflammation, lipid and glucose metabolism, and insulin signaling at the molecular and epigenetic levels [25, 34].

We found that only-SFA associated CpGs localized in the gene bodies and intergenic regions, with lower proportions in the near and far transcription sites (Fig. 2). DNA methylation in the gene body region has been previously found to display a direct effect on gene expression [35]. Accordingly, DNA methylation changes in adipose tissue resulting from gastric bypass surgery have been reported to be mostly located in CpGs concentrated in the body and intergenic regions [36]. In summary, we suggest that the SFA-related differences in DNA methylation in relation to genetic location may link to epigenetic regulation. Additionally, to rule out the possibility of that a SNP on the CpG site would confound our results, the USCS genome browser was searched based on genomic coordinates for single nucleotide polymorphism (SNP). We found only one SNP (rs138784380—genomic deletion) that was on the corresponding CpG site (cg05921947, *ADCY2*).

The CpGs from which methylation was specifically associated with liver SFA, and not with NASH, were enriched for neuroactive ligand–receptor interaction, morphine addiction, and retrograde endocannabinoid signaling pathways (Table 2). The role of endocannabinoids and related signaling molecules in controlling food intake and satiety is well established [37–39]. This is in line with the findings that many studies have identified that dietary FA composition differentially affects appetite and acts as endocannabinoid receptor ligands, with the SFAs being on the harmful end of the spectrum [40–42]. Thus, our current results highlight the probable involvement of SFAs in the regulatory mechanisms linked with hunger and energy intake. The effects of dietary fatty acids are heterogeneous in terms of metabolic and physiologic outcomes that are most correctly measured as diet-induced thermogenesis (DIT) and energy expenditure (EE) [43]. Dietary SFAs are linked with more weight gain compared to other FAs, attributed to the fact that SFA is more favored in the body as a storage nutrient since it is oxidized slower than other FAs [43]. It is very interesting to note that high-fat diet, specifically SFA, has been found to be related to development of obesity, without ingestion of too many calories, indicating a direct effect on feeding efficiency [43]. It has been also suggested that hepatic neuronal network is a major player in the regulation of calorie intake and energy expenditure, hence affecting the feeding efficiency [44]. Furthermore, alterations in hepatic sympathetic neuronal activity have been linked with obesity and NAFLD [45, 46]. Although our results concentrating on SFAs and liver we cannot speculate the mechanisms regulating energy metabolism centrally, our findings suggest a connection between SFAs, satiety, and NAFLD development.

Our results indicate that the 54 genes annotated to only-SFA CpGs also play a major role in maintaining the metabolic functions of the liver, mainly by regulating glucose and insulin metabolism (Fig. 3). For example, the gene *CACNA1B* codes for a calcium ion channel that regulates glutamate transport across the cell membrane, which is a major substrate for glucose production. There were many genes coding for G-proteins and their subunits, such as *GNAI3, GNGT2, GNG12, GABRB1, GABRP,* and *GABRD*. We agree that many of these, such as *GABRB1, GABRP,* and *GABRD* genes, are not known to be major regulators in the liver. Most of them are essential for a variety of cellular functions, and few of these had been identified to be major players in NAFLD progression [47–49]. We also identified a key gene, *PRKCA*, characterized as a lipid-dependent kinase with a major role in both positive and negative modulation of insulin action [50]. Additionally, we observed DNA methylation of *TSPO,* encoding a potential molecular imaging biomarker for noninvasively distinguishing NAFLD, [51] to be related with SFA and associated with *TSPO* mRNA expression. Finally, we acknowledge that many of the genes related to identified CpG sites are primarily identified as neuronal genes, and thus, their role in liver metabolism remains unclear.

We also found DNA methylation of another ten of these CpGs correlated with the mRNA expression of their neighboring genes (Table 3). Most of the identified genes were involved in functions related to neurotransmission and substrate metabolism, such as *PRKAA1*, *RAC1*, *SDHAF2*, *C14orf132*, *FCHSD2*, *GALE*, and *ZDHHC4*. These genes have previously been found to be key players in controlling energy metabolism and appetite by actively sensing and responding to stimuli, most importantly to the nutrients [52–57]. Interestingly, we found, *KDM5B*, a gene encoding lysine-specific histone demethylase that belongs to the jumonji/ARID domain-containing family of histone demethylases. In general, histone methylation plays an important role in epigenetic regulation of gene expression and hence might be of biological relevance in the case of SFA. Overall, these results reflect that SFAs seem to influence the gene expression changes of important genes by affecting the DNA methylation.

The interesting novel finding was that fasting plasma glucose levels were more often correlated with the SFA-related CpGs, while for the only-NASH CpGs the most significant associations were with fasting serum insulin levels (Fig. 4). Accordingly, we have previously shown that NASH-related epigenetic alterations associate with changes in insulin action, [30] Contrary to what was expected based on previous studies, [58, 59], we did not find any significant change in the proportions of the only-SFA associated CpGs and cholesterol and/or LDL-c levels. Nonetheless, the percentage of significantly associated CpGs for HDL-c and TG levels were significantly higher for only-SFA, as compared to the coverage on the whole 450 K array (Fig. 4).

We found palmitic and stearic acid to be the most correlated more often with DNA methylation of the CpGs linked with SFA. Earlier studies in humans and human-derived cells have found that both palmitic and stearic acids actively interact with the epigenome [25, 27, 29, 60]. For instance, excess palmitic and stearic acid is known to induce inflammation and metabolic dysregulations in various cell models (including primary murine hepatocytes) along with alterations in histone acetylation and DNA methylation [61–64]. Our findings reiterate that among the individual SFAs, both palmitic and stearic acids are the main contributors to the observed SFA-related correlation with DNA methylation.

We acknowledge that the current study is cross-sectional and, hence, limits our conclusions related to causation. However, it is unethical to have follow-up liver biopsies in these types of studies. In addition, the study size is limited and as all the subjects were morbidly obese, we cannot generalize the results to lean or normal-weight subjects. However, with this unique dataset with liver samples collected during the bariatric surgery from a gender-, BMI-, and age-matched study cohort, we were able to have a holistic view of the liver, monitoring simultaneously histology, DNA methylation, and gene expression.

## Conclusions

In conclusion, we propose that one of the mechanisms how SFA may contribute to the metabolic dysregulation in NAFLD might occur at the level of DNA methylation. In addition, we suggest that liver SFA-related differences in DNA methylation profile may contribute more to hyperglycemia, while insulin-related differences are more linked to changes in methylation specific to NASH in this particular study population and setting. Due to the cross-sectional study design, we fully admit no conclusions about causality are possible. Further research is also needed to elucidate the mechanistic links of our current findings.

## Methods

### Study participants and analyses of clinical and metabolic parameters

Participants were selected from an ongoing Kuopio Obesity Surgery (KOBS) Study [65]. Fifty-one individuals undergoing laparoscopic Roux-en-Y gastric bypass (LRYGB) operation (mean ± SD, 49.3 ± 8.5 years old; BMI, 43.7 ± 6.2 kg/m^2^; 19 males) were included. All individuals had their anthropometric and biochemical evaluations done before the surgery. Fasting serum samples were subjected to lipid profiling and insulin measurements, while fasting plasma glucose levels were measured, as described before [66]. Type 2 diabetes was defined according to World Health Organization (WHO) criteria. The study was performed in accordance with the Declaration of Helsinki. Written informed consent was obtained from all participants, and the study protocol was approved by the Ethics Committee of the Northern Savo Hospital District (54/2005, 104/2008, and 27/2010).

### Liver histology and liver phenotype

Liver biopsies were obtained with ultrasonic scissors during the elective LRYGB operation from all the patients participating in the KOBS study. The overall histological assessment of liver biopsies was performed by a pathologist according to the standard criteria [67, 68]. Individuals were then grouped into one of the three categories: 1. Normal liver without any steatosis, inflammation, ballooning, or fibrosis, 2. SS (steatosis > 5% without evidence of hepatocellular ballooning, inflammation, or fibrosis), or 3. NASH (detailed in Table 1) [65, 66].

### Liver total SFA content using NMR spectroscopy

Fasting concentrations of liver SFA content were analyzed by proton nuclear magnetic resonance (NMR) spectroscopy in native liver samples. At first, the liver samples were homogenized, mixed, sonicated, centrifuged, and dried. Prior to NMR analysis, the extracted lipids were redissolved into 600 µl of CDCl_3_ containing 0.03% of tetramethylsilane as a reference substance. ^1^H NMR spectra of extracted lipids were recorded on a Bruker Avance III HD 600 NMR spectrometer with acquisition time of 5 s and the relaxation delay of 15 s, as described previously [66]. The PERCH NMR software was used for all the lineshape fitting analyses.

### Quantification of individual SFAs using gas chromatography

The liver fatty acid composition in triglycerides (TG), cholesteryl esters (CE), and phospholipids (PL) was measured by gas chromatography (GC). The liver fatty acids were analyzed according to previously described methods [66]. In short, liver lipids were extracted with chloroform–methanol (2:1), and the lipid fractions were separated by solid phase extraction with an aminopropyl column. Fatty acids in TG, CE, and PL were transmethylated with boron trifluoride in methanol and were analyzed with a 7890 A gas chromatograph (Agilent Technologies, Inc., Wilmington, DE, USA) equipped with a 25-m FFAP column using nonadecanoic acid as the internal standard as detailed before [66]. The total SFAs in each of these fractions (molar percentages, mol%) were then correlated with the liver total SFA content identified using the NMR spectroscopy (Additional file 4: Fig. S3). The TG fraction was found to be most strongly correlated (*r* = 0.35, *p* value < 0.05), and the individual SFAs from this fraction were considered for further analyses.

### DNA methylation and gene expression in human liver

The DNA extracted from the human liver biopsies was used for DNA methylation analysis using Infinium HumanMethylation450 BeadChip (Illumina, San Diego, CA, USA). The raw methylation data in *β*-values were converted to *M*-values for bioinformatic and statistical analyses; however, *β*-values were used for describing the data and creating figures as reported earlier along with the methodology [30, 69]. DNA methylation of 455,526 CpGs was associated with liver SFA content. RNA expression was analyzed in liver samples from a subset of individuals included in this study (*n* = 20) using the HumanHT-12 Expression BeadChip (Illumina). The array covers 28,688 coding transcripts, and all the procedures were performed in accordance with the manufacturer’s recommendations and are detailed elsewhere [69]. A total of 12,064 transcripts passed the quality control filter and were considered for further analysis.

### Identification of SFA-related DNA methylation sites including genomic enrichment analysis

To identify differences in DNA methylation in the liver associated with liver total SFA content, a linear regression model was used including gender, BMI, and age as covariates and DNA methylation as the dependent variable. To account for multiple testing in the genome-wide analysis, we applied false discovery rate (FDR) as well as Bonferroni correction and considered significant probes with *q*-value < 0.05.

We overlapped the identified CpGs with the ones we had previously found to be associated with NASH [30], to get three distinct datasets, only-NASH, NASH-SFA, and only-SFA CpGs. Comparison of these three datasets to annotated function categories, including relation to genes (within 1500 bp of a transcription start site [TSS], 200 bp of a TSS, a 5′ untranslated region [UTR], first exon, gene body, 3′UTR, and intergenic) and CpG islands (Island, shore, shelf, open sea), was performed using UCSC (University of California Santa Cruz) Genome Browser annotations supplied by Illumina. A *χ*^2^ test of independence with 1° of freedom was used to determine whether there was evidence of enrichment for any of these groups as compared to the coverage on the 450 K BeadChip. (*p* value < 0.01 was considered statistically significant.)

### Pathway analysis of genes mapped to CpG sites associated with liver SFA

We performed a KEGG pathway analysis using the WebGestalt tool [70] to identify biological pathways enriched for genes mapped to CpGs associated with liver SFA (*q*-value < 0.05). For this, we considered the only-SFA and NASH-SFA groups separately. StringDB (Version 11.5) was used to visualize the gene interactions for the enriched pathways and selection of the genes [31]. The top genes with the maximum interaction nodes or involvement in all the enriched pathways were shortlisted and discussed. We also correlated these top-ranking CpGs with the mRNA expression of their nearby gene(s) (within the *cis* distance 500 kb upstream and 100 kb downstream of the gene, Additional file 5: Table S2), and *p* value < 0.05 was considered statistically significant.

### Statistical analyses

Clinical data are presented as mean ± SD or median (interquartile range: IQR). One-way ANOVA (continuous variable) or *χ*^2^ test (categorical variable) was used to study the differences in the clinical variables, liver histology, and total SFA content for the three study groups (normal liver, simple steatosis (SS), and NASH; *n* = 51). After applying further post hoc Bonferroni correction for multiple testing, *p* values < 0.05 were considered significant.

Pearson correlation analysis was used to correlate DNA methylation of selected CpGs with gene expression, clinical variables, and individual SFAs (14:00-myristic acid, 15:00-pentadecanoic acid, 16:00-palmitic acid, 17:00-heptadecanoic acid, and 18:00-stearic acid). For these analyses, a *p* value < 0.05 was considered statistically significant. For the correlations with clinical variables, a *χ*^2^ test of independence with 1° of freedom was used to determine if the proportion of sites was significantly (*p* value < 0.05) different for each variable as compared to the coverage on the 450 K BeadChip.

## Supplementary Information

**Additional file 1:** **Table S1**. SFA-related DNA methylation in human liver. The table contains the 3169 CpGs found to be associated with total liver saturated fat content.

**Additional file 2**: **Fig. S1**. Correlation between the DNA methylation of CpGs and clinical variables. The heatmaps represent Pearson’s correlation coefficient for each clinical variable when correlated with the DNA methylation of the CpGs. The three heatmaps correspond to each of the three groups as shown on the *y*-axis, the most prominent associations with fasting plasma glucose and fasting serum insulin are highlighted with a red box. All the data were checked for normality and values were log-transformed, if required. The percentages on the top of each column represent the percentage of significant associations for the corresponding clinical variable. The significant correlations are with *p* value < 0.05.

**Additional file 3**: **Fig. S2**. Correlation between the DNA methylation of CpGs and individual SFA. The heatmaps represent Pearson’s correlation coefficient for individual SFA (mol%) when correlated with the DNA methylation of the CpGs. The three heatmaps correspond to each of the three groups as shown on the *y*-axis. The percentages on the top of each column represent the percentage of significant associations for the corresponding SFA. The significant correlations are with *p* value < 0.05.

**Additional file 4**: **Fig. S3**. Correlation between the NMR total SFA content and GC-derived liver total SFA (mol%) for different fractions. The scatter plot represents the correlation between the NMR-derived liver total SFA content (*x*-axis) and GC-derived liver total SFA (mol%, *y*-axis). The equations for corresponding groups and the *R*^2^ value are displayed on the graph. The blue dots represent the cholesteryl esters (CE) fraction, yellow dots are for phospholipids (PL) and red ones are for triglycerides (TG) fraction.

**Additional file 5**: **Table S2**. Correlation between the top-ranking CpGs and the mRNA expression of their nearby gene(s). The mRNA expression of neighboring genes within the cis distance 500 kb upstream and 100 kb downstream of the gene.

**Additional file 6**: **Table S3**. Proportions of significant correlation between the CpGs and the clinical variables. The correlations for all the four groups CpGs were correlated with the clinical variables and were adjusted for multiple testing. The proportions of significant correlations (adjusted *p* value < 0.1) for each variable and group are tabulated in the file.

**Additional file 7**: **Table S4**. Proportions of significant correlation between the CpGs and the individual fatty acids. The correlations for all the four groups CpGs were correlated with the individual fatty acids and were adjusted for multiple testing. The proportions of significant correlations (adjusted *p* value < 0.1) for each variable and group are tabulated in the file.

## Data Availability

The datasets supporting the conclusions of this article are included within the article and its additional files.

## Abbreviations

BMI: Body mass index
CpGs: CpG sites
FDR: False discovery rate
FFAs: Free fatty acids
GC: Gas chromatography
HDL-c: High-density lipoprotein cholesterol
KEGG: Kyoto Encyclopedia of Genes and Genomes
KOBS: Kuopio Obesity Surgery
LDL-c: Low-density lipoprotein cholesterol
LRYGB: Laparoscopic Roux-en-Y gastric bypass
NAFLD: Non-alcoholic fatty liver disease
NMR: Nuclear magnetic resonance
NASH: Non-alcoholic steatohepatitis
*PRKCA*: Protein kinase C alpha
SD: Standard deviation
SFAs: Saturated fatty acids
SS: Simple steatosis
TG: Triglycerides
*TSPO*: Translocator protein
TSS: Transcription start site
