# Supplementary figures and images for: Liver saturated fat content associates with hepatic DNA methylation in obese individuals
Source: Clin Epigenetics. 2023 Feb 11;15:21. doi: 10.1186/s13148-023-01431-x (PMC9921201; doi:10.1186/s13148-023-01431-x)

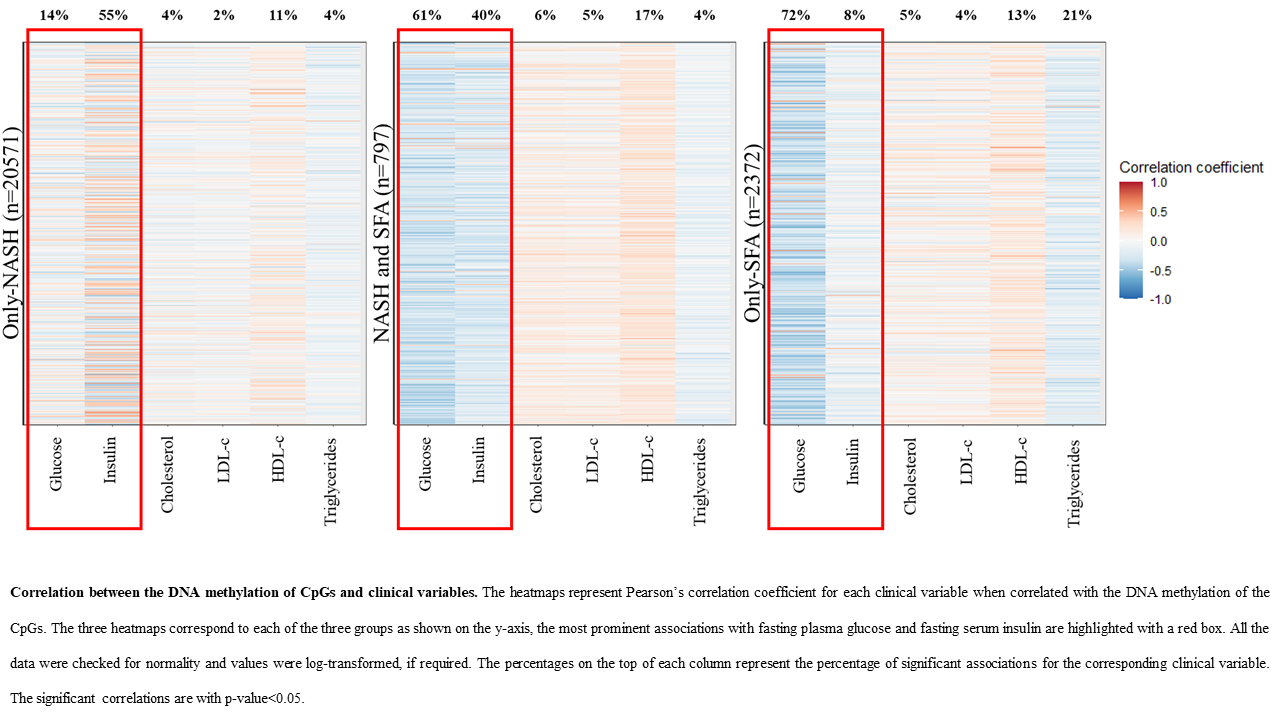

Supplement: Supplementary file 2 — Additional file 2: Fig. S1. Correlation between the DNA methylation of CpGs and clinical variables. The heatmaps represent Pearson’s correlation coefficient for each clinical variable when correlated with the DNA methylation of the CpGs. The three heatmaps correspond to each of the three groups as shown on the y-axis, the most prominent associations with fasting plasma glucose and fasting serum insulin are highlighted with a red box. All the data were checked for normality and values were log-transformed, if required. The percentages on the top of each column represent the percentage of significant associations for the corresponding clinical variable. The significant correlations are with p value < 0.05. [file 13148_2023_1431_MOESM2_ESM.tif]

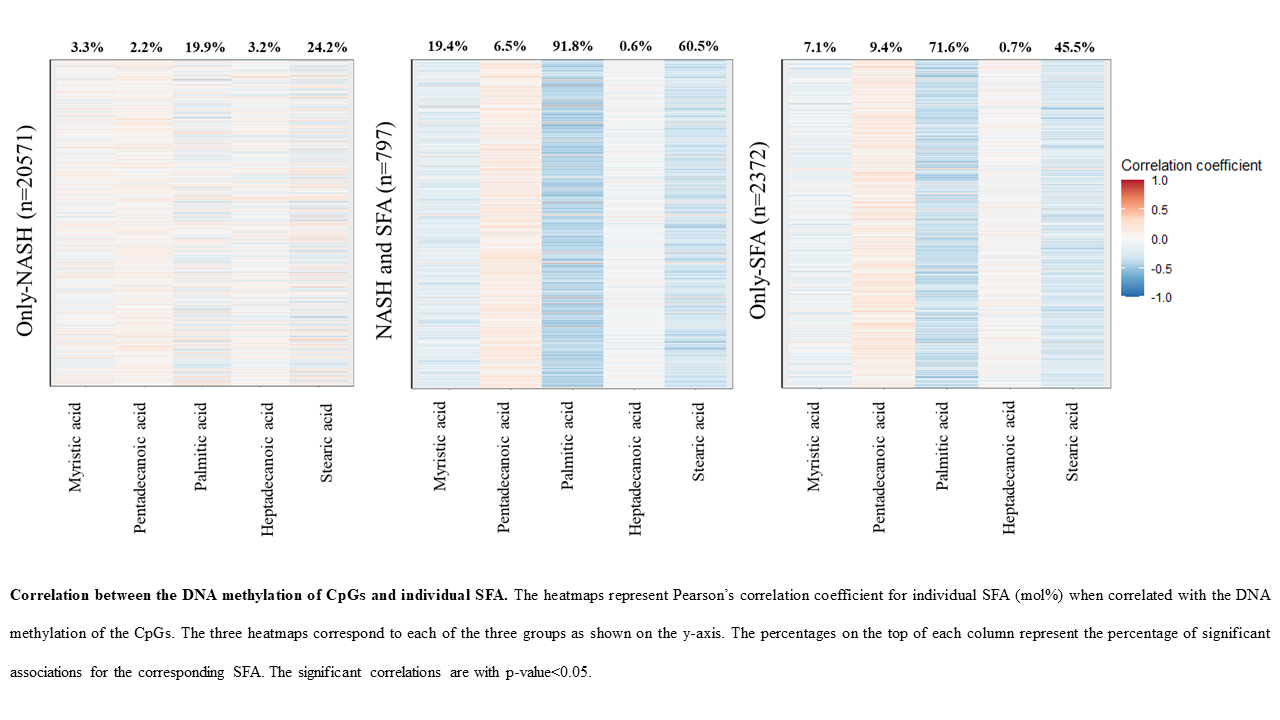

Supplement: Supplementary file 3 — Additional file 3: Fig. S2. Correlation between the DNA methylation of CpGs and individual SFA. The heatmaps represent Pearson’s correlation coefficient for individual SFA (mol%) when correlated with the DNA methylation of the CpGs. The three heatmaps correspond to each of the three groups as shown on the y-axis. The percentages on the top of each column represent the percentage of significant associations for the corresponding SFA. The significant correlations are with p value < 0.05. [file 13148_2023_1431_MOESM3_ESM.tif]

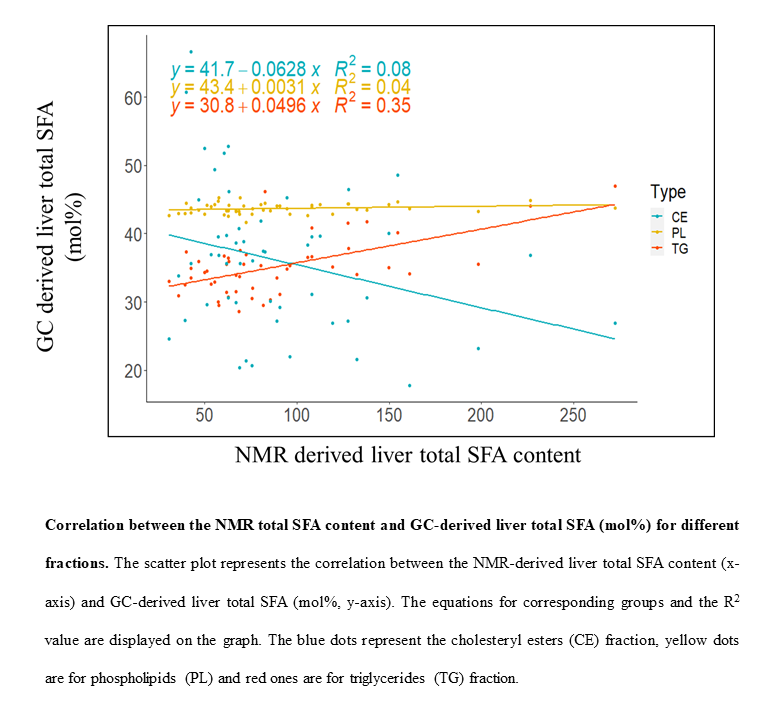

Supplement: Supplementary file 4 — Additional file 4: Fig. S3. Correlation between the NMR total SFA content and GC-derived liver total SFA (mol%) for different fractions. The scatter plot represents the correlation between the NMR-derived liver total SFA content (x-axis) and GC-derived liver total SFA (mol%, y-axis). The equations for corresponding groups and the R2 value are displayed on the graph. The blue dots represent the cholesteryl esters (CE) fraction, yellow dots are for phospholipids (PL) and red ones are for triglycerides (TG) fraction. [file 13148_2023_1431_MOESM4_ESM.tif]
